# Evaluating the Safety and Efficacy of Intravenous Thrombolysis in Acute Ischemic Stroke Patients Without Perfusion Deficit: A Retrospective Analysis

**DOI:** 10.3390/brainsci15101034

**Published:** 2025-09-24

**Authors:** Omar Alhaj Omar, Stefan T. Gerner, Slava Alikevitch, Samra Hamzic, Maxime Viard, Anne Mrochen, Priyanka Böttger, Martin Juenemann, Tobias Braun

**Affiliations:** 1 Department of Neurology, Justus-Liebig-University, Klinikstrasse 33, 35392 Giessen, Germanysamra.hamzic@neuro.med.uni-giessen.de (S.H.); martin.juenemann@neuro.med.uni-giessen.de (M.J.);; 2 Translational Neuroscience Network Giessen (TNNG), 35392 Giessen, Germany; 3 Center of Mind, Brain & Behavior (CMBB), 35032 Marburg, Germany; 4 Department of Neurology, University Hospital Erlangen, 91054 Erlangen, Germany; 5 Department of Neurology, Kantonsspital Winterthur, 8401 Winterthur, Switzerland; 6 Department of Cardiology, Angiology and Critical Care Medicine, Justus-Liebig-University, 35390 Giessen, Germany; 7 Department of Neurology, Lahn-Dill-Kliniken Wetzlar, 35578 Wetzlar, Germany

**Keywords:** acute ischemic stroke, CT perfusion, intravenous thrombolysis

## Abstract

**Background/Objectives:** Acute ischemic stroke (AIS) remains a major cause of morbidity and mortality worldwide. Although advanced imaging modalities, such as CT perfusion (CTP), are increasingly being used in clinical decision-making, the necessity and added value of perfusion imaging prior to intravenous thrombolysis (IVT) within early time windows remains uncertain. We aim to evaluate the safety and functional outcomes of IVT in AIS patients without perfusion deficits on CTP. We question the requirement of perfusion mismatch for IVT eligibility and hypothesize that IVT is safe and beneficial even in the absence of a perfusion deficit. **Methods:** A retrospective analysis was conducted using data from the Giessen Stroke Registry, focusing on AIS patients who underwent CTP imaging and received IVT between 01/2018 and 12/2020. Patients who underwent endovascular therapy were excluded. Clinical data, including demographics, National Institutes of Health Stroke Scale (NIHSS) scores, modified Rankin Scale (mRS) scores, and complications, were collected. Patients were dichotomized based on the presence of perfusion lesions and compared in terms of efficacy outcomes (i.e., NIHSS or mRS improvement during the hospital stay) and safety outcomes (i.e., post-thrombolytic hemorrhagic complications). **Results:** Of the 89 AIS patients with available CTP data who received IVT, 34 (38%) had a perfusion deficit and 55 (62%) did not. There were no significant differences between the groups in terms of hemorrhagic complications or functional outcomes at discharge (NIHSS and mRS). Clinical improvement from admission to discharge was similar in both groups. **Conclusions:** Our findings suggest that IVT is safe and clinically effective even in AIS patients without detectable perfusion deficits on CTP within the standard therapeutic window. These results support current guideline recommendations that do not mandate perfusion imaging for early presenters. Routine use of CTP in this context may be of limited clinical utility and could unnecessarily delay treatment or introduce additional risks in the first 4.5 h.

## 1. Introduction

Acute ischemic stroke (AIS) is one leading cause of morbidity and mortality worldwide [1]. The diagnosis of ischemic stroke has become more established over the last few years. The acute management of ischemic stroke within the recommended therapeutic time window of 4.5 h is a well-established practice and should be implemented promptly, provided that contraindications have been thoroughly assessed and excluded. Early treatment, particularly with reperfusion therapies, is essential to preserve brain tissue and optimize neurological recovery [2]. Contemporary guidelines also emphasize not delaying IVT to obtain advanced imaging in otherwise eligible early-window patients [3,4].

The advent of mismatch imaging has enabled identification of the ischemic penumbra, brain tissue that may still be salvaged through recanalization therapy even in later time windows than 4.5 h. This supports the “tissue window” concept, which allows treatment eligibility to extend beyond strict temporal constraints [5,6,7]. However, CT perfusion imaging may not always reveal a perfusion deficit, even in symptomatic patients. In such cases, intravenous thrombolysis (IVT) remains indicated within the time window and should not be withheld based on imaging findings alone [8]. Most of these patients exhibit minor yet functionally disabling deficits [9], and early neurological deterioration is not uncommon, often resulting in poor outcomes [10,11,12]. Furthermore, lacunar ischemia can be overlooked on perfusion imaging, despite a severe clinical presentation [13]. In the last two years, studies of wake-up/unknown-onset stroke and imaging selection (e.g., DWI–FLAIR and perfusion imaging) have expanded the evidence base, but have also highlighted variability across centers [14]. In the early treatment window (≤4.5 h), however, the incremental value of perfusion imaging for IVT decision-making remains uncertain.

IVT is a well-established and highly effective treatment strategy for patients with AIS [15,16]. Despite advancements in imaging techniques, the necessity of perfusion imaging to guide IVT remains controversial. Although perfusion imaging helps identify salvageable brain tissue in the extended time window, some patients with minor strokes may not exhibit a clear perfusion deficit upon CT perfusion despite having significant clinical symptoms. In these cases, withholding IVT due to a lack of apparent mismatch could potentially result in missed opportunities for functional recovery. Moreover, emerging comparative data suggest non-contrast CT scan (NCCT) ±CT-Angiographic-anchored workflows can achieve outcomes comparable to perfusion-selected pathways in routine practice, underscoring the risk that additional imaging may delay treatment in the early window [17]. Given that early neurological deterioration can occur in minor strokes and lead to long-term disability, prompt treatment remains essential, and the central controversy—guideline-driven rapid IVT versus imaging-gated selection—remains unresolved in early presenters. This emphasizes the need for further research into IVT efficacy in this subgroup [18]. Accordingly, we asked: Is perfusion imaging necessary in early-window IVT decision-making?

The safety of IVT for patients without a perfusion deficit is another critical consideration. Studies have demonstrated that IVT carries a small but well-characterized risk of hemorrhagic transformation, as well as other hemorrhagic complications such as intracranial hemorrhage and systemic bleeding [19]. However, whether the absence of a perfusion deficit meaningfully alters this risk profile—particularly in early presenters—has not been clearly defined in the recent literature [20].

We aim to assess the clinical efficacy and safety of IVT in early time-window stroke patients without perfusion deficits upon CT perfusion and to determine whether safety and efficacy profiles differ based on the presence or absence of perfusion lesions. By explicitly addressing this gap, our study contributes contemporary data to the debate over whether perfusion imaging is required to guide IVT decisions within the ≤4.5 h window.

## 2. Materials and Methods

### 2.1. Patient Selection and Study Design

The local ethics committee of the Justus-Liebig University Giessen, Germany, approved this retrospective study under reference number 220/21. We screened the Giessen Stroke Registry (ClinicalTrials.gov Identifier: NCT05295862) for patients with AIS and who underwent CT perfusion imaging and recanalization therapy of IVT or endovascular therapy (EVT). We included all subjects who received only IVT from 01/2018 and 12/2020. Exclusion criteria were: (i) endovascular therapy (EVT); and (ii) missing baseline imaging (non-contrast CT and/or CT perfusion), typically in external referrals whose initial imaging was not retrievable in our PACS. No cases were excluded for severe motion artefact. All included patients had complete clinical records for the predefined outcomes.

### 2.2. Data Acquisition

Clinical data were retrieved from the retrospective institutional database and included the following parameters: patients’ demographics, prestroke-modified Rankin scale, modified Rankin scale on discharge, comorbidities, mode of admission and National Institute of Health Stroke Scale (NIHSS) on admission and at discharge [21]. Further, we assessed laboratory results and etiology according to TOAST criteria [22].

### 2.3. Neuroimaging

According to our institutional standard operating procedure, all patients presenting with a disabling neurological deficit within a 24 h time window routinely undergo CT perfusion imaging upon admission. Imaging was performed using a dual-source CT scanner (SOMATOM Force^®^, Siemens, Erlangen, Germany) with multimodal CT, including CT angiography and CT perfusion. Regarding IVT, we administered thrombolysis after the initial native CT scan using exclusively rt-PA in the established dosage according to the patients’ estimated body weights [23].

A multimodal CT imaging protocol, consisting of CT angiography and CT perfusion, was performed immediately thereafter. The infarct core was defined as brain regions with markedly reduced cerebral blood volume (CBV) and cerebral blood flow (CBF), indicating irreversible tissue damage. ‘‘Penumbra’’ refers to areas with prolonged mean transit time (MTT) or time-to-maximum (T-[max]) perfusion parameters (>6 s delay according to our protocol) [24,25] but with preserved CBV, indicating potentially salvageable tissue at risk of infarction. Mismatch is defined as the difference between the volume of the penumbra and the infarct core volumes, representing the volume of at-risk but viable brain tissue. In our study, we used a qualitative visual mismatch depending on consultant neuroradiologist’s experience when the area of prolonged perfusion (as seen on MTT or T-[max] maps) is substantially larger that the infarct core. Perfusion status was classified qualitatively in routine clinical workflow by one consultant neuroradiologist and one radiology resident; readers were not blinded to clinical information, and discrepancies were resolved by discussion. Although we did not capture a formal inter-rater reliability coefficient in this retrospective cohort, we recognize its importance and note that standardized assessment and prospective agreement metrics should be incorporated in future studies.

### 2.4. IVT Prrotocol

Adults with a clinical diagnosis of acute ischemic stroke causing a disabling neurologic deficit were eligible for intravenous thrombolysis if treatment could be initiated ≤4.5 h from last-known-well, or for unknown-onset/wake-up strokes when selected by imaging (CT perfusion mismatch imaging) per institutional policy. Patients treated in the extended time window (>4.5 h from last-known-well, including wake-up strokes) were selected for IVT according to our institutional protocol. Specifically, IVT was administered when CT perfusion demonstrated a perfusion deficit consistent with a penumbra–core mismatch; in cases without a mismatch, IVT was considered when the neurologic deficit was disabling and the baseline non-contrast CT showed no demarcation of infarction. Non-contrast CT was required to exclude intracranial hemorrhage and large established infarction, and blood pressure was controlled to <185/110 mmHg prior to the thrombolytic bolus. The thrombolytic agent was alteplase 0.9 mg/kg (maximum 90 mg; 10% IV bolus followed by the remainder as a 60 min infusion). Contraindications (treated as absolute unless local policy stated otherwise) included intracranial hemorrhage on imaging or clinical suspicion of subarachnoid hemorrhage; intracranial/intraspinal surgery or serious head trauma within the recent past (commonly ≤3 months); active internal bleeding or known bleeding diathesis; platelet count <100 × 10^9^/L; current anticoagulation with INR >1.7, markedly elevated aPTT, or DOAC use within 48 h without a drug-specific level confirming no activity; persistent severe uncontrolled hypertension (>185/110 mmHg) despite therapy; arterial puncture at a noncompressible site within the last 7 days; and infective endocarditis. Correctable hypoglycemia (<50 mg/dL) was treated and patients were reassessed before lysis.

### 2.5. Outcomes

As a primary outcome parameter, we defined safety of IVT (i.e., frequency of hemorrhagic complications according to the Heidelberg bleeding classification [HI indicates hemorrhagic infarctions and PH indicates parenchymal hematomas]) [26]. We compared the rates of parenchymal bleedings of classes PH1 and PH2, intraventricular hemorrhage, and subarachnoid hemorrhage among all patients.

As secondary outcomes, we defined (i) the mRS at discharge (ii) NIHSS at discharge, (iii) in-hospital mortality (iv) NIHSS improvement on discharge (defined as NIHSS improvement of ≥4 points or reaching **0–1**) (v) mRS improvement on discharge (≥1-point reduction in mRS or achieving the baseline mRS), and (vi) favourable outcome on discharge (i.e., mRS 0–2). Two independent raters blinded to all clinical data conducted the radiological assessments. In cases of initial disagreement, a consensus rating was reached.

### 2.6. Statistical Analysis

Statistical analysis was conducted using SPSS Version 29. Distribution of data was analyzed using the Kolmogorov–Smirnov test. Data with normal distribution are presented as mean ± standard deviation (SD) and compared using Student’s *t*-test. Data without normal distribution are presented as median and range and compared using the Mann–Whitney U test. Categorical variables were presented as frequency and percentage, and groups were compared using Pearson chi square or Fisher’s exact test. All statistical tests were two-sided, and the significance level was set at α = 0.05. A logistic regression analysis was conducted to assess the effect of the variable ‘‘time window > 4.5 h, unclear onset, or wake-up stroke’’ on the primary and secondary outcome parameters by comparing patients with and without perfusion deficits. Given the retrospective, single-center design and limited sample size (*n* = 89), the study was not powered for precise effect estimation; accordingly, our analyses are descriptive and hypothesis-generating, and we do not present effect sizes or confidence intervals in the main manuscript.

## 3. Results

**Figure 1:**
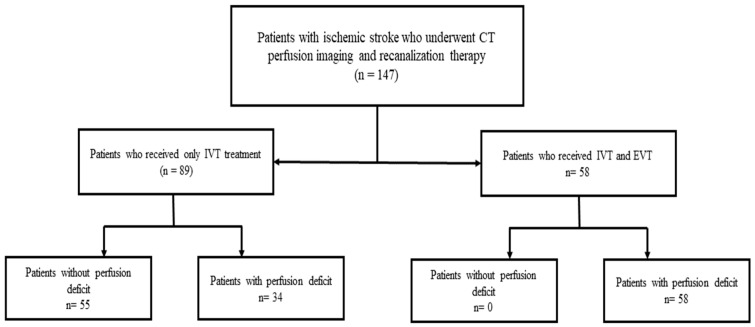
Flow chart of study cohort.

During the study period, 147 patients with acute ischemic stroke underwent CT perfusion imaging and received recanalizing therapy (see Figure 1). After exclusion of 58 patients who underwent EVT, 89 patients with IVT only were available for analyses.

Baseline and stroke characteristics of patients dichotomized based on the presence of perfusion deficit are presented in Table 1. Of 89 patients, perfusion deficits were detected in 34. The group of patients with a perfusion deficit were predominantly male, with females comprising only 20.6%. Other demographic characteristics showed no significant differences between the two groups. Length of hospital stay was significantly longer in patients with perfusion deficit. Risk factor profiles were comparable, but arterial hypertension was more frequent in those without perfusion deficits (83.6% vs. 73.5%; *p* = 0.254). Regarding the mode of presentation, all patients with a perfusion deficit were transported by emergency medical services whereas a proportion of patients without a perfusion deficit presented as walk-ins. Patients without a perfusion deficit exhibited longer prehospital delays, with a mean onset-to-door time of 128 min. Among those with an extended time window (>4.5 h), unclear symptom onset, or wake-up strokes, 5 patients (14.7%) demonstrated a perfusion deficit whereas 4 patients (7.3%) did not. There were no significant differences in door-to-needle times between the two groups.

No significant differences were observed between the two groups regarding baseline clinical status at admission, as assessed using mRS and NIHSS.

Early ischemic changes on initial non-contrast CT—including hyperdense artery sign, loss of the insular ribbon, obscuration of the lentiform nucleus, cortical sulcal effacement, subtle parenchymal hypodensity, and loss of gray–white differentiation—were identified in 3 patients (8.8%) with a perfusion deficit, but in none of the patients without a deficit. No established infarctions were detected on baseline imaging in either group. A perfusion mismatch was present in all patients with a perfusion deficit (100%). A follow-up cerebral MRI during hospitalization was performed in 41.2% of patients with a perfusion deficit and in 58.2% of those without.

Regarding the primary outcome, no significant differences were observed between the groups (refer to Table 2). A symptomatic intracerebral hemorrhage (sICH) occurred in 4 of 34 patients (11.8%) with a perfusion deficit and in 4 of 55 patients (7.3%) without.

The incidence of parenchymal hemorrhage (PH1 or PH2) was low in both groups: 2.9% (1/34) in patients with a perfusion deficit and 1.8% (1/55) in those without. Specifically, PH1 occurred in 2 patients (5.9%) with a perfusion deficit and in 3 patients (5.5%) without. No cases of PH2 or subarachnoid hemorrhage were observed in either group. An intraventricular hemorrhage occurred in one patient from each group (2.9% vs. 1.8%; *p* = 0.477).

With respect to stroke etiology, patients without a perfusion deficit most commonly exhibited microangiopathic infarctions (41.8% vs. 5.9%; *p* ≤ 0.001), whereas macroangiopathic strokes were more frequently observed in the group with a perfusion deficit (38.2% vs. 14.5%; *p* = 0.011). Discharge status and stroke-related complications did not differ significantly between the groups. No significant differences were observed between the two groups regarding patient status at admission, as measured using mRS NIHSS. Across primary and secondary endpoints, we did not observe a consistent directional trend; absolute between-group differences were small and bidirectional (e.g., sICH 11.8% vs. 7.3%; PH1/PH2 5.9% vs. 5.5%; favorable outcome 79.4% vs. 78.2%).

There were no significant differences between the two groups in terms of discharge status or stroke-related complications.

## 4. Discussion

Our findings suggest that IVT is safe and effective in AIS patients even in the absence of a perfusion deficit. This aligns with current guidelines recommending IVT based on clinical presentation within therapeutic window rather than solely on imaging findings [4,27]. A novel aspect of our study is the focus on an IVT-only cohort within the ≤4.5 h window—including patients without measurable perfusion deficits—providing pragmatic, real-world evidence for decision-making when perfusion maps are negative.

Given that only 9 patients in our cohort either were outside the recommended therapeutic time window or presented with wake-up strokes, the sample size is too small to allow for definitive conclusions or recommendations regarding the use of IVT in this specific subgroup. Current international guidelines consistently recommend IVT for eligible patients presenting within the established time window, and importantly, they do not mandate perfusion imaging in such scenarios [3,4,28]. In our exploratory analysis of this small subgroup, we found no statistically significant differences in either the primary or secondary outcome measure. This aligns with the rationale underlying current guideline recommendations: that perfusion imaging within the therapeutic window offers limited incremental value in guiding treatment decisions. Indeed, the core clinical decision—whether to administer IVT—is already adequately supported by clinical assessment and standard imaging in patients presenting within this window. Consistent with this, we did not observe a coherent directional trend favoring either group across endpoints; absolute differences were small and bidirectional, supporting a cautious interpretation.

Although perfusion imaging may offer valuable insights in selected cases—particularly in those with wake-up strokes or unclear symptom onset—its routine use in all patients within the time window appears questionable. The evidence supporting the added value of perfusion imaging in the early hours is still limited, as can be seen in the relatively sparse literature on this topic [25,29,30]. In fact, our findings indirectly support this position: the use of perfusion imaging in these patients did not yield any additional clinically relevant information. Therefore, omitting nonessential perfusion studies in this context could reduce patient exposure to ionizing radiation and contrast agents, which is a meaningful consideration in clinical practice [31,32]. Our data therefore speak to a practical question clinicians face: when perfusion maps are negative but the deficit is disabling, IVT can be pursued without clear evidence of added risk.

Etiologically, microangiopathic strokes were significantly more common in patients without a perfusion deficit, whereas macroangiopathic strokes were predominant in those with a perfusion deficit. Despite these differences in stroke subtype, functional outcomes at discharge, as measured using the mRS and NIHSS were similar between the two groups, reinforcing the clinical benefit of IVT irrespective of perfusion status.

Importantly, safety outcomes, including rates of SICH, IVH, SAH and PH1/PH2, were similar between groups. The observed hemorrhage rates align with established data from major stroke trials, suggesting that IVT does not impose a higher risk of bleeding complications in patients without perfusion deficits [33,34]. These findings support the continued use of IVT in eligible patients with minor strokes, particularly given the risk of early neurological deterioration in this population [35].

Ultimately, larger, well-designed studies are needed to determine the specific clinical scenarios in which perfusion imaging may offer significant benefit. Until then, our data reinforce the current paradigm that standard clinical and imaging evaluation remains sufficient for decision-making within the therapeutic time frame. Future work should also standardize perfusion assessment and prospectively measure inter-rater reliability to reduce classification variability.

Our results align with contemporary guideline recommendations that prioritize clinical presentation and non-contrast CT in the early window and are consistent with observational data showing that perfusion imaging does not uniformly change IVT decisions within ≤4.5 h [4,24,25,26,27]. The similar functional and safety outcomes we observed between patients with and without perfusion deficits mirror prior cohort reports and large registries that documented low rates of hemorrhagic complications under protocolized IVT [30,31,32]. Taken together, these data suggest that, while perfusion imaging is valuable in extended or uncertain time windows, its incremental utility for routine early-window IVT selection may be limited.

This study is retrospective and single-center, introducing potential selection and information bias. Although we included consecutive IVT-only patients, external referrals with missing baseline imaging could not be adjudicated centrally. The sample size is modest, and the extended-window/wake-up subgroup is small, limiting power for between-group comparisons and increasing imprecision. Residual confounding (e.g., stroke etiology, prehospital delay, blood pressure control, and center-specific practice patterns) cannot be excluded despite standardized protocols. Perfusion-status classification relied on our institutional workflow, including qualitative visual mismatch, which may differ from quantitative thresholds used elsewhere. Outcomes were assessed at routine clinical time points (including discharge), which may not fully capture longer-term recovery; long-term outcomes (e.g., 90-day mRS) were not available in this retrospective cohort, which limits conclusions about sustained efficacy; future studies should incorporate standardized follow-up. We did not collect radiation- or contrast-related complications; while CTP generally entails higher radiation dose and contrast volume than NCCT (±CTA), our study cannot estimate this risk. The cohort size also limits power to detect small differences in door-to-needle time. Finally, findings pertain to IVT-only management and may not generalize to settings with broader use of EVT or alternative thrombolytics. In terms of impact on interpretation, the modest sample and retrospective design increase the risk of type II error (i.e., missing modest differences), the qualitative, non-blinded perfusion classification may attenuate true associations via misclassification, and discharge-only outcomes could underestimate late recovery or delayed complications; collectively, these factors likely bias results toward the null and argue for cautious inference.

## 5. Conclusion

Routine perfusion imaging should not delay IVT in eligible patients presenting within ≤4.5 h; clinical assessment plus non-contrast CT is sufficient for early-window decision-making. Perfusion imaging can be reserved for extended or uncertain onset cases and specific diagnostic questions. Prospective, adequately powered trials are warranted to refine these indications. As noted, such trials should incorporate standardized perfusion metrics, blinded assessments with reliability reporting, and 90-day outcomes to clarify longer-term effectiveness.

## Figures and Tables

**Table 1:** Baseline and clinical characteristics.

	With Perfusion Deficit (*n* = 34)	Without Perfusion Deficit (*n* = 55)	*p*
**Demographics**			
**Female sex ^b^**	7 (20.6)	25 (45.5)	0.018 *
**Age ^a^**	67.8 (12.4)	69.8 (14.4)	0.503
**BMI ^a^**	28.4 (4.6)	27.6 (4.2)	0.385
**Baseline characteristics and Risk factors**			
**Length of stay (days) ^a^**	15.9 (16.5)	10.7 (4.6)	0.028 *
**Number of risk factors ^c^**	2 (1;3)	2 (1;3)	0.425
**Atrial fibrillation ^b^**	9 (26.5)	8 (14.5)	0.168
**Arterial hypertension ^b^**	25 (73.5)	46 (83.6)	0.254
**Diabetes ^b^**	11 (32.4)	9 (16.4)	0.081
**Hypercholesteremia ^b^**	8 (23.5)	14 (25.5)	0.84
**Smoking ^b^**	7 (20.6)	12 (21.8)	0.896
**Previous stroke ^b^**	7 (20.6)	12 (21.8)	0.896
**Coronary heart disease ^b^**	9 (26.5)	10 (18.2)	0.359
**Peripheral arterial disease ^b^**	1 (2.9)	3 (5.5)	0.583
**Art of Presentation**			
**Presentation through emergency medical service ^b^**	32 (94.1)	47 (85.5)	0.214
**Self presentation ^b^**	0 (0.0)	4 (7.3)	n.a.
**Presentation from peripheral hospital ^b^**	2 (5.9)	4 (7.3)	0.394
**Process times**			
**Time since onset of symptoms (min) ^a^**	92.0 (44.3)	128.8(77.7)	0.039 *
**Time window > 4.5 h or unclear onset or wake-up stroke ^b^**	5 (14.7)	4 (7.3)	0.263
**Door to needle time (min) ^a^**	53.2 (20.7)	45.8 (19.8)	0.101
**Status before and at admission**			
**mRS before admission ^c^**	0 (0;0)	0 (0;0)	0.386
**mRS at admission ^c^**	3 (2;4)	3 (2;4)	0.623
**NIHSS at admission ^c^**	4 (3;6)	4 (3;6)	0.733
**Radiologic data**			
**early ischemia ^b^**	3 (8.8)	0 (0)	n.a.
**brain infarction ^b^**	0 (0)	0 (0)	n.a.
**Perfusion-Mismatch ^b^**	34 (100)	n.a.	n.a.
**cMRT during hospital stay ^b^**	14 (41.2)	32 (58.2)	0.122
**SICH ^b^**	4 (11.8)	4 (7.3)	0.477
**Etiology (TOAST)**			
**Macroangiopathy ^b^**	13 (38.2)	8 (14.5)	0.011 *
**Microangiopathy ^b^**	2 (5.9)	23 (41.8)	<0.001 *
**Cardiac embolic ^b^**	10 (29.4)	10 (18.2)	0.222
**Others ^b^**	2 (5.9)	1 (1.8)	0.311
**Cryptogenic ^b^**	7 (20.6)	13 (23.6)	0.741
**Status at discharge and complications of strokes**			
**Aspiration pneumonia ^b^**	6 (17.6)	8 (14.5)	0.700
**Epileptic seizure ^b^**	1 (2.9)	1 (1.8)	0.743
**mRS at discharge ^c^**	1 (0;2)	1 (0;2)	0.800
**NIHSS at discharge**	1 (0;3)	0 (0;2.5)	0.689
**mRS improvement ^b^**	26 (76.5)	39 (70.9)	0.571
**NIHSS improvement ^b^**	57 (62.0)	41 (74.5)	0.882
** Favorable outcome ^b^**	27 (79.4)	43 (78.2)	0.892
**In-hospital mortality ^b^**	1 (2.9)	2 (3.6)	0.871

^a^ Mean ± SD. ^b^ *n* (%). ^c^ Median (interquartile range: 25th–75th percentile). * statistically significant.

**Table 2:** Analysis of outcomes.

	With Perfusion Deficit (*n* = 34)	Without Perfusion Deficit (*n* = 55)	*p* Value
**SICH ^b^**	4 (11.8)	4 (7.3)	0.732
**HI1 and HI2 ^b^**	1 (2.9)	1 (1.8)	0.732
**PH1 and PH2 ^b^**	2 (5.9)	3 (5.5)	0.933
**SAH ^b^**	0 (0.0)	0 (0.0)	n.a.
**IVH ^b^**	1 (2.9)	1 (1.8)	0.477

^b^ *n* (%).

## Data Availability

The data presented in this study are available on request from the corresponding author. The data are not publicly available due to privacy and ethical restrictions associated with patient information.
